# Totally implantable venous access devices: retrospective analysis of different insertion techniques and predictors of complications in 796 devices implanted in a single institution

**DOI:** 10.1186/1471-2482-14-27

**Published:** 2014-05-08

**Authors:** Elisa Granziera, Marco Scarpa, Angelo Ciccarese, Bogdan Filip, Matteo Cagol, Valentina Manfredi, Rita Alfieri, Connie Celentano, Sandra Cappellato, Carlo Castoro, Muzio Meroni

**Affiliations:** 1 Anestesiology Unit, Veneto Institute of Oncology (IOV-IRCCS), Padova, Italy; 2 Surgical Oncology Unit, Veneto Institute of Oncology (IOV-IRCCS), Padova, Italy; 3 Department of Surgery, Iasi University Hospital, Iasi, Romania

**Keywords:** Totally implantable venous access device, US guided, Chemotherapy

## Abstract

**Background:**

The aim of this study was to assess the efficacy and safety of totally implanted vascular devices (TIVAD) using different techniques of insertion.

**Methods:**

We performed a retrospective study using a prospective collected database of 796 consecutive oncological patients in which TIVADs were inserted. We focused on early and late complications following different insertion techniques (surgical cutdown, blind and ultrasound guided percutaneous) according to different techniques.

**Results:**

Ultrasound guided technique was used in 646 cases, cephalic vein cutdown in 102 patients and percutaneous blind technique in 48 patients. The overall complication rate on insertion was 7.2% (57 of 796 cases). Early complications were less frequent using the ultrasound guided technique: arterial puncture (p = 0.009), technical failure (p = 0.009), access site change after first attempt (p = 0.002); pneumothorax occurred in 4 cases, all using the blind percutaneus technique. Late complications occurred in 49 cases (6.1%) which required TIVAD removal in 43 cases and included: sepsis (29 cases), thrombosis (3 cases), dislocation (7 cases), skin dehiscence (3 cases), and severe pain (1 case).

**Conclusion:**

Ultrasound guided technique is the safest option for TIVAD insertion, with the lowest rates of immediate complications.

## Background

The use of totally implantable venous access devices (TIVADs) has changed the care and quality of life for cancer patients treated with chemotherapy. TIVADs represent a convenient option when long-term venous access is indicated, particularly for administration of cytotoxic medications or intravenous targeted agents in cancer patients over a long period of time [1]. The current debate regarding TIVAD utilisation in clinical practice includes the access site (internal, external jugular and subclavian vein) and the insertion technique (open, percutaneous or with ultrasound guidance). Cephalic vein approach has the advantage of safety and low incidence of early complications [2] but it is affected by a high rate of failure. Subclavian vein catheters are located in an easy accessible area but they are affected by a relatively high risk of thrombosis, vein stenosis, catheter fatigue and they have the highest risk of pneumothorax at insertion [3]. The internal jugular approach is the preferred approach for tunnelled infusion catheter approach with the lowest incidence of venous thrombosis [4]. Usually percutaneus techniques through the Seldinger technique are preferred [5-7] but in some cases surgeons prefer the open approach in the cephalic or subclavian vein. Early complications include: pneumothorax, hemothorax, air embolism, accidental arterial puncture, cardiac arrhythmia, pericardial tamponade and brachial plexus injury [8,9]. Late complications include: bloodstream infection, thrombosis, catheter dysfunction, rupture, migration or embolisation, "pinch-off" syndrome, superior vein cava erosion and perforation, extravasation, pocket infection and port inversion [10-12]. The refinement of the technique and the implanted devices led to a decreased rate of potential life threatening complications.

The aim of this study was to evaluate the short and long term outcomes after TIVAD implantation in a, consecutive series of patients by comparing the different insertion techniques.

## Methods

### Study design

The study was performed according to the second principles of the Declaration of Helsinki. The study was retrospectively reviewed and approved by the Ethical Committee of the Veneto Institute of Oncology (protocol number 0001897; Internal code: 2014/13/NOTIFICA; date of approval 24th February 2014). This study is a retrospective review of a prospectively collected database created in 2006 by the authors including 796 consecutive totally implantable venous access devices (TIVAD) applied from November 2006 to November 2011 in the Surgical Oncology Unit of the Veneto Institute of Oncology (IOV-IRCCS). Follow up continued until the device was removed, patient died or the study was closed (30.11.2011). Patients gave their informed consent to have their data collected in a database and to have them anonymously used for scientific purposes. The devices were implanted in adult patients affected by several different neoplastic diseases requiring chemotherapy. All patients had the same type of TIVAD implanted: an M.R.I. Implantable Port with open end 8 French polyurethane single lumen venous catheter (C. R. Bard, Inc. Murray Hill, New Jersey, USA). The main tumor types were gastrointestinal, breast, head and neck, ovarian and sarcoma.

Preoperative evaluation included medical history and physical examination, focusing on possible anatomic pitfalls (cervical or mediastinal adenopathy, chest wall tumours, previous neck or thoracic surgery), body habitus, previous vascular access placements and complications. Laboratory studies consisted in full blood count and coagulation. Exclusion criteria were platelet count < 80.000/mm^3^, INR > 1.5, neutrophil count <1000/mm^3^, fever or sepsis. During the pre-treatment visit patients were informed on the procedure, risks, benefits, data management and follow up, and written informed consent for the inclusion in this study was obtained.

### Data collection and follow-up

A score of procedural difficulty was assigned to each TIVAD implantation, ranging from 1 (easy) to 4 (extremely difficult). Patient characteristics, which included diagnosis, indication for catheter placement, age, height, weight, results of laboratory test parameters and current medications were recorded. Device type, site of venous access, surgeon and anaesthesiologist performing the procedures and placement complications were documented. Data from hospital admissions and telephone follow-up were recorded at regular intervals and prospectively collected in a database. All device-associated complications were recorded during follow up. Complications were classified into two main categories: early (intraoperative and post-implantation period to first use) and late complications (occurring after the first chemotherapy course administered through the device).

Criteria for the diagnosis of device-related bacteraemia were defined as follows: a over 10-fold increase in colony-forming units (CFU) of bacteria per ml of blood obtained through the device in comparison to peripheral blood cultures; over 1000 CFU of bacteria obtained through the device, in the absence of peripheral blood cultures; or positive catheter tip culture upon removal in the appropriate clinical setting. Port pocket infection was defined by induration, erythema and tenderness around the port with culture-positive material aspirated from the port pocket. Cutaneous site infection was defined by induration, erythema, or tenderness and exudate at the port surface needle access site. Thrombosis was detected with ultrasound and/or venography when clinically suggested by progressive arm or facial swelling.

The following assumptions were used as the basis for determining the cost-effectiveness of each strategy. Operative room hourly costs were based on current estimates of standard charges in an Italian setting (2013), including salaries for a surgeon, a anesthesiologist and two nurses (650 euro per hour). We calculated the overall cost for our institution.

### Implantation techniques

Devices were implanted in the operating room, using maximal sterile-barrier precautions, under local anaesthesia, administering a mixture of Lidocaine 1% and Ropivacaine 0.5% and employing fluoroscopic control. Premedication with midazolam 0.01-0.035 mg/kg (1–3 mg) was provided in case of patient anxiety. A single dose of Cefazoline 2 g was administered intravenously before the procedure. In beta-lactam allergic patients, Vancomycin 15 mg/kg was used. In order to prevent clot formation and catheter blockage, TIVAD were flushed with 20 ml saline and then filled with 5 ml of a solution containing 50 U/ml heparin. Standard protocol was to flush all the devices with heparinized saline solution after use and on a monthly outpatient basis.

Three different techniques were used for implantation. From November 2006 until January 2008 devices were placed using either a cephalic vein cut-down or a blind percutaneous approach based on anatomic landmarks. The choice of insertion technique was at the discretion of the surgeon. Since January 2008 the percutaneous approach was improved using ultrasound guidance.

### Cut-down approach

A single incision was made in the upper anterior chest wall along the delto-pectoral groove as first described by Heimbach and Ivey [13]. The cephalic vein was identified and isolated between 2 vessel loops. The catheter was then directly inserted into the cephalic vein through a transverse venotomy. The distal cephalic vein was tied off. The reservoir was inserted superficially into a subcutaneous pocket, with the device located just inferior to the skin incision. Fluoroscopy was used to confirm positioning of the tip of the catheter at the cavo-atrial junction. No additional postoperative radiographs were performed.

### "Blind" percutaneous technique

The preferred access site was the subclavian vein (SV). The internal jugular vein (IJV) was chosen when SV cannulation was contraindicated for anatomic pitfalls. A Seldinger technique was used to access the vein with dilators and peel away sheaths for the insertion. In case of subclavian vein cannulation, the site of puncture was at the inferior border of the clavicle, between the middle and the lateral third of it, directed toward the fingertip pressed firmly into the suprasternal notch. The needle passed beneath the inferior margin of the clavicle in a horizontal plane and directed toward the anterior margin of the trachea at the level of the suprasternal notch. The internal jugular vein was cannulated at the top of the triangle between the sternal and clavicular head of the sternocleidomastoid muscle, advancing the needle through the skin at a 45° angle in the direction of the omolateral nipple. Fluoroscopy confirmed the position of the catheter at the cavo-atrial junction, and a completion upright chest radiograph was performed before leaving the surgical ward to assess for the presence of pneumothorax. Usually 2 incisions were necessary: a small incision at the catheter exit site from the skin and a second larger incision for location of the implantable access device. A subcutaneous tunnel was made to pass the catheter from 1 incision to the other.

### Ultrasound guided percutaneous technique

A 7–12 MHz linear-array ultrasound probe connected to a real-time ultrasound unit (General Electric Logiq® P5, GE Healthcare Clinical Systems SrL,), and focused at 2–4 cm depth, was covered with ultrasonic gel and wrapped in a sterile plastic sheath. Standard ultrasound two-dimensional (2D) imaging was used to measure the depth and calibre of the IJV or SV, evaluate its patency and compressibility. In cases of pre-existing thrombus formation and/or failure to gain access due to trauma or other anatomical anomalies, the IJV or SV on the controlateral side was catheterised. Catheterisation was performed under continuous dynamic observation of real-time 2D images. The in-plane approach was used to achieve a long-axis view of the needle, allowing full visualization of the shaft and tip of the needle. The low lateral approach, as described by Jernigan and modified by Pittiruti [14], was used to obtain cannulation of the distal IJV, or whenever possible, of the brachiocephalic vein. The individuation of the brachiocephalic vein was obtained giving to the ultrasound probe a caudal direction, placing it with an inclination of 20–30 degrees with the neck and the clavicle. The ultrasound-guided subclavian vein catheterisation was performed positioning the probe below the clavicle, usually in the lateral third, to obtain a long axis of the vein. Using an in plane approach, the SV was cannulated just medially to the junction of cephalic vein to the axillary vein. An 18-gauge, 7-cm needle was advanced through the skin under ultrasound guidance into the vein. A guidewire was then placed through the needle into the vein, and the needle was removed. Fluoroscopy confirmed the position of the catheter at the cavo-atrial junction, considering an optimal position when the catheter tip was located within a range of 2 cm at the lower border of the right main bronchus. Also in this case, 2 incisions were necessary: 1 small incision at the wire exit site from the skin and a second larger incision for location of the implantable access device. A subcutaneous tunnel was made to pass the catheter from 1 incision to the other. No additional postoperative radiographs were performed except in case of repeated attempts or procedural complications.

### Statistical analysis

Statistical analysis was performed using both Microsoft Excel and STATISTICA 7.1 software (Statsoft, Tulsa, OK, USA). Continuous data are expressed as medians and interquartile ranges, while dichotomic data are expressed as frequencies and proportions. Continuous data were compared with two tailed Mann-Whiteny U test or Kruskall-Wallis ANOVA where appropriate, while frequencies were compared with Fisher's exact test or Chi square analysis. Odds ratios were calculated to assess the risk of complication. Only predictors resulting significant at univariate analysis were included in multivariate models. Life table analyses were created to assess risk rate of TIVAD removal. Two-tailed p-values <0.05 were considered significant.

## Results

### Patient characteristics

Overall characteristics of the study population and divided by the insertion technique are shown in Tables 1 and 2. The majority of patients had solid organ cancer; only 29 (3.64%) had hematologic malignancies (lymphoma). The median follow up was 35.1 (23.0-55.4) weeks. During the period of our study three different implantation techniques were used. In the first part of our study, we utilised the surgical cutdown or blind percutaneus according to surgeon and anestesiologist preference. In the second part of our study, all the consecutive TIVADs were inserted using the ultrasound technique. The anatomical choice for venous access is shown in Table 3. In the surgical cutdown technique, the cephalic vein was used in almost all cases (92/102 patients), in the rest of the cases (10 patients) the external jugular vein was used. The access site for blind cutaneus technique was the subclavian vein in 41 cases or the jugular vein in 5 cases (2 cases missing). The majority of the access sites were, the distal internal jugular vein or brachiocephalic vein through the supraclavicular approach (515/646 patients), then the subclavian vein (29 cases) or the distal internal jugular vein (100 cases).

**Table 1:** Characteristics of the study population and follow up

**Characteristic**	**Total population 796 pts**
Age (years, median/range)	61 (21–87)
Gender (F/M)	432/364
Body Mass Index (kg/m^2^, median, range IQR)	24.23 (21.9-27.18)
Follow up (weeks, median, range IQR)	35.14 (23–55.4)
Follow up completed (n° pts/total pts)	535/796
Length of use (weeks, median, range IQR)	22.71 (10.1-41.9)
Time of in-situ permanence (weeks, median, range IQR)	33.71 (15.71-57)
Pancreatic cancer (n° pts)	20
Colon/rectal cancer	218
Gastric cancer	67
Oesophageal cancer	102
Lymphoma	29
Breast cancer	127
Brain cancer	14
Head and neck cancer	33
Sarcoma	48
Liver cancer	15
Lung cancer	36
Ovarian cancer	38
Melanoma	13
Urothelial cancer	6
Teratoma	5
Other miscellaneous	25
White blood cells count (cells ×10^9^/L, median, range IQR)	6 (1.98-8.77)
Hb (g/L, median, range IQR)	12 (10–13)
Platelets (cells × 10^9^/L, median, range IQR)	276 (223–341.5)
PT (%, prothrombin time, median, range IQR)	88 (75–100)
PTT (sec, partial thromboplastin time, median, range IQR)	28 (26–30)
INR (international normalized ratio, median, range IQR)	1,00 (0.5-1.12)

**Table 2:** Characteristic of patients divided in groups according to the implantation technique (Intention To Treat)

**Characteristic**	**(SC)**	**(BP)**	**(US)**	**p- value**
	**(102 pts)**	**(48 pts)**	**(646 pts)**	
Age (years, median/range)	60	61	61	0.478
Gender (M/F)	52/50	15/33	297/349	0.07
Body Mass Index (kg/m^2^, median, range IQR)	25.3 (22.4-27.3)	28.3 (24.9-30.9)	24.2 (21.9-28.8)	0.003
Follow up (weeks, median range IQR)	81 (66–94)	106 (98–118)	31 (20–46)	<0.001
Follow up completed (n° pts/total pts)	76/102	31/48	413/646	0.288
Time of in-situ permanence (weeks, median, range IQR)	38.8 (25.1-57.8)	70.6 (51.6-96.2)	25.3 (13.1-47.1)	0.001
Operating time (min, median, range IQR)	45 (40–60)	45 (40–60)	30 (20–40)	<0.001
Costs (euro median IQR)	487.5 (429–650)	487.5 (429–650)	325 (214.5-429)	<0.001
Score of difficulty (median range IQR,)	1 (1–2)	1 (1–1)	1 (1–1)	0.046

Surgical Cutdown: SC; Blind Percutaneus technique: BP; US guided Percutaneous technique: US.

**Table 3:** Anatomic site for venous access

**Successful vein accessed**	**Number**	
Cephalic vein	Right	19
	Left	74
Proximal internal jugular vein (cervical access)	Right	68
	Left	39
Subclavian vein	Right	16
	Left	65
Distal internal jugular vein/brachiocephalic vein (supraclavicular access)	Right	306
	Left	209
Total		796

### Clinical outcome

In 102 patients placement of TIVAD via the cephalic vein cutdown approach was initially attempted, 87 patients underwent successful insertion while 15 required conversion to a percutaneous approach (success rate 85.3%), 11 using the blind technique, 3 using the ultrasound guided technique. In one patient, it was impossible to access any central vein due to the anatomical pitfalls and a peripheral inserted central venous access (PICC) was inserted under ultrasound guidance. Lack of success was related to small size of vein (n = 6) or inability to locate the vein (n = 8) or impossibility to transverse the angle of insertion of cephalic vein into the subclavian vein (n = 1). The percutaneous blind technique was initially used in 48 patients, and was successful in 45 patients (93.7%), 3 patients required conversion to a surgical approach. Lack of success was related to occurrence of pneumothorax attempting subcavian vein cannulation (n = 2) or inability to locate the vein (n = 1). The ultrasound guided approach was used as a primary technique in 646 patients with a success rate of 99.7% (644/646). In 13 patients, a second attempt changing puncture site was necessary, while 2 patients required conversion to a surgical approach because of fibrotic or collapsed vein. Mean operating time was 51 minutes for cephalic vein cutdown approach, 49 minutes for blind percutaneous technique and 33 minutes for ultrasound guided technique.

The cost, expressed as cost of time of use of the operative room of US-guided TIVAD placement, was significantly lower compared to cutdown approach and to percutaneous blind technique. In fact, in spite of the use of the same device, the duration of the procedure was significantly shorter in patients who had a US guided TVAD placement.

The overall early complication rate including changing the puncture site using then same technique was 7.2% (57 of 796 cases) as listed in Table 4. The only severe intraoperative complications requiring immediate treatment were three of the four pneumothoraxes and one of the two arrhythmias (a supraventricular paroxistic tachycardia requiring pharmacological treatment). All the other complications did not require any treatment except for a longer monitoring before discharge. The technique failure was significantly reduced using ultrasound compared with the surgical technique (p < 0,001). The occurrence of pneumothorax and arterial puncture were significantly higher in the landmark group as compared to the ultrasound group. Interestingly, our data shows a significantly increased number of sepsis in the surgical group compared with those documented in the ultrasound group, although the overall rate of late complications was significantly lower in this group.

**Table 4:** Early complications divided in groups according to the implantation technique (Intention To Treat)

**Complication**	**Surgical cutdown (102 pts) SC**	**Blind percutaneous (48 pts) BP**	**US guided percutaneous**	**Fisher exact test p value**	**Fisher exact test p value**	**Fisher exact test p value**
			**(646 pts) US**	**SC vs BP**	**BP vs US**	**SC vs US**
**Pneumothorax**	0 (0%)	4 (8.3%)	0 (0%)	**0.009**	**<0.001**	NA
**Arterial puncture**	0 (0%)	4 (8.3%)	8 (1.2%)	**0.009**	**0.006**	0.390
**Arrhythmia**	0 (0%)	1 (2.1%)	2 (0.3%)	0.301	0.193	0.179
**Technique failure**	15 (14.7%)	3 (6.2%)	2 (0.3%)	0.181	**0.002**	**<0.001**
**Access site change after first attempt**	0 (0%)	5 (10.4%)	13 (2%)	**0.002**	**0.005**	0.233
**Total**	15 (14.7%)	17 (35.4%)	25 (3.9%)	**0.005**	**<0.001**	**<0.001**

**Figure 1:**
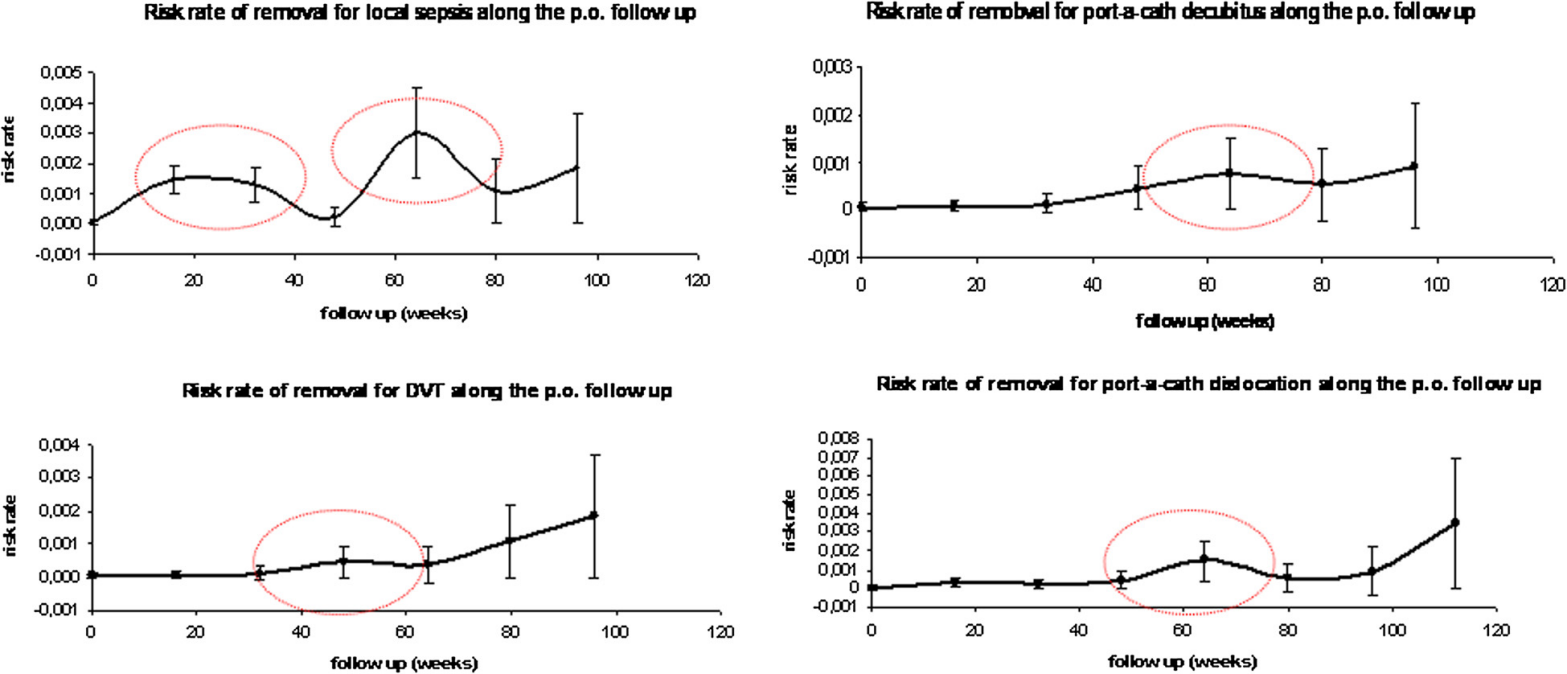
Risk analysis for TIVAD urgent removal.

**Figure 2:**
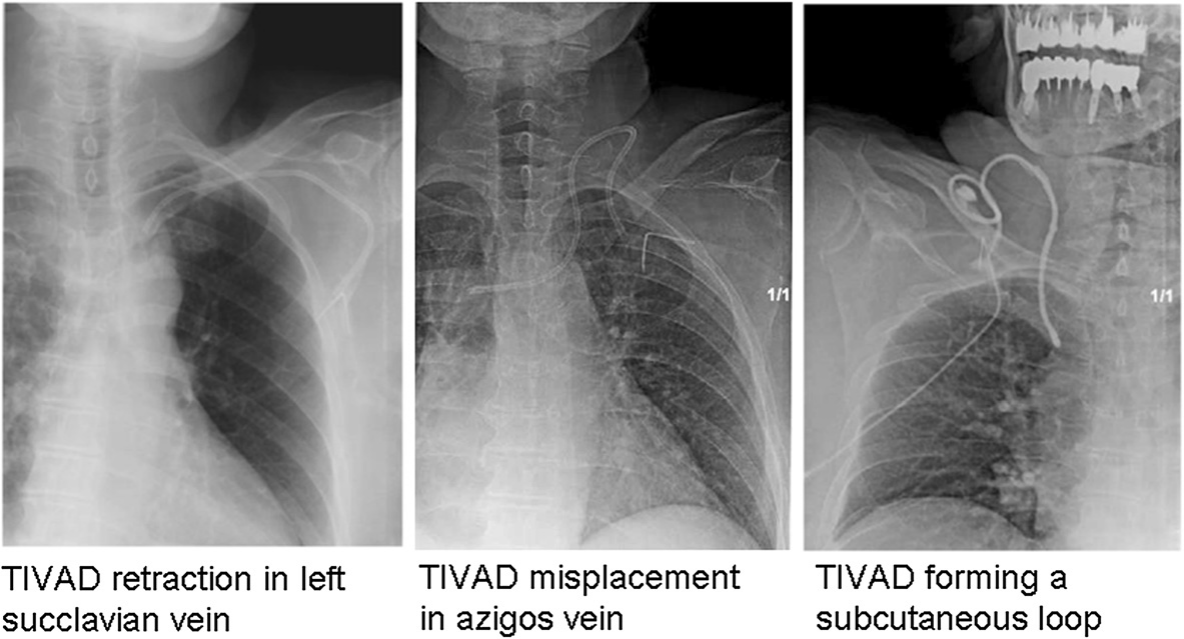
Late TIVAD misplacement that did not compromise their function.

Late complications occurred in 49 of 796 patients (6.1%), requiring removal of the TIVAD in 43 (5.2%) as listed in Table 5. The microorganisms isolated in TIVAD related sepsis were candida glabrata, candida parapsilosis, bacillus species, streptococcus mitis, pseudomanas aeruginosa, and staphylococcus epidermidis. The other cases of late complications that did not require removal of the TIVAD were: one case of sepsis treated with intravenous antibiotics, one thrombosis that required anticoagulants with remission, one malfunction, 2 cases of extravasation or severe pain. As shown in Table 6, we performed a logistic regression for prediction of late complications that lead to TIVAD removal. Only the open approach and low levels of white blood cells resulted to be independent predictor of urgent TIVAD removal. Risk rate analysis of TIVAD removal for sepsis showed a peak between 20 and 30 weeks and a second one after 60 weeks DVT, decubitus and dislocation had a risk rate peak after 60 weeks. Risk analysis for TIVAD urgent removal is shown in Figure 1. Late misplacement of TIVAD occurred but they did not require any further intervention unless it precluded its function. Some misplacement cases are shown in Figure 2.

**Table 5:** Late complications divided in groups according to the implantation technique (Intention to treat)

	**Surgical cutdown**	**Blind percutaneous**	**US guided percutaneous**	**Fisher exact test p value**	**Fisher exact test p value**	**Fisher exact test p value**
	**(102 pts) SC**	**(48 pts) BP**	**(646 pts) US**	**SC vs BP**	**BP vs US**	**SC vs US**
Complication	Removal	Removal	Removal			
Sepsis	11 (10.8%)	0 (0%)	18 (2.8%)	0.016	0.392	<0.001
Thrombosis	2 (1.9%)	0 (0%)	1 (0.1%)	0. 560	0.999	0.050
Dislocation	1 (0.9%)	2 (4.1%)	4 (0.6%)	0.240	0.058	0.999
Dehiscence	0 (0%)	0 (0%)	3 (0.5%)	NA	0.998	0.999
Severe pain	0 (0%)	0 (0%)	1 (0.1%)	NA	0.999	0.999
Total	14 (13.7%)	3 (6.2%)	26 (4.0%)	0.269	0.713	<0.001

**Table 6:** Predictors of late complications

**Variable**	**Predictors**	**Odds ratio**	**95% CI**	**p-value**
Removal for sepsis	Surgical isolation	2,706	0,9955 - 7,3546	0,051
p = 0,0838	Current CT	0,799	0,3102 - 2,0599	0,643
	BMI	1,002	0,9940 - 1,0109	0,570
	Head and neck cancer	3,045	0,6287 - 14,7470	0,167
	WBC count	0,817	0,6682 - 0,9976	0,047
Removal for DVT	Anticoagulant or antiaggregant therapy	16,410	0,5533 - 486,6924	0,106
p = 0,0273	Surgical isolation	45,218	1,5944 - 1282,4410	0,026
Removal for dislocation	US guided puncture	0,320	0,0699 - 1,4626	0,142
p = 0,2947	Difficult puncture	1,318	0,5202 - 3,3380	0,561

## Discussion

Different and long term chemotherapy treatments or nutritional support require a long term venous access in a cancer patient. Since 1982, the increasingly frequent use of TIVADs has reduced the burden of peripheral vein complications and the restriction on physical activities of patients and, thus, improving quality of life. On the other hand, there is a strict association between the incidence of TIVAD-related complications and the occurrence of psychosocial complaints (eg, depression, fatigue, social impairment, and decreased quality of life) [15]. Ideally, TIVAD positioning should have a low rate of morbidity after insertion and in the long term follow up, and should be easily tolerable for the patient during his daily routine. In our retrospective series, three different implantation techniques were used and compared in order to identify the technique that may guarantee the best outcome.

In our series, the overall complication rate is consistent with data reported by several studies [4,16-18], that range between 2 to 14.4%. Furthermore, similarly to recent series, we observed a decreasing trend for early complications. Rare major complication as described in previous studies or case series reports, such as hemothorax [19], air embolism [20], pericardial tamponade [21] and brachial plexus injury [22] did not occur in our series. At the beginning of our experience, the surgical cutdown technique in cephalic vein was used and it had the advantage of no early complications but at the price of a higher technical failure rate. Nevertheless, the complications rate did not seem to be correlated with the experience of the operator [6,7,23]. In our series, 4 cases of pneumothoraxes occurred after the blind percutaneous technique and, three of them needed to be treated with a chest drain tube. Pneumothorax seems to be the most frequent and important complication after insertion, with an immediate clinical impact. Its incidence varies between 0.5-6% [24], highly correlated with the blind subclavicular approach and with the multiple attempts of needle passage. In our series, US-guided insertion completely eliminated this complication probably because it provides a direct visualization of the needle and all the underlying anatomical structures during the whole procedure. Although accidental arterial puncture seems to occur mainly during subclavicular approach with a frequency between 6-8% [8], in our series we observed 8 accidental artery puncture of the ultrasound guided approach group occurred during the "learning curve" period of the technique (first 3 months). However, they did not require any treatment except for a longer monitoring before discharge.

According to the results of this study, the occurrence of these complications was reduced by limiting the number of needle passages with ultrasonic identification of the vein. Indeed, this technique permitted to obtain a significantly lower rate of technical failure without changing the access site for TIVAD implantation. A further benefit from two dimensional US guidance for central venous access compared with the other methods was a faster access. In fact, the operating time was significantly shorter in the ultrasound group compared to the others. As demonstrated in a meta analysis investigating ultrasound guidance [25], data reflected on cost effectiveness. The resource saving is obtained by shortening the time spent by clinicians and nurses to achieve successful cannulation and to deal with complications. This time saving procedure reduced the use of expensive operative room time.

In our series, surgical cutdown technique in cephalic vein and low levels of white blood cells resulted to be a significant predictor of TIVAD removal for complication. In the series described by Mansfield et al., the factor associated to early complications after TIVAD placement are prior attempts or catheterisation of the vein, local modification due to previous radiotherapy, a high body mass index and multiple needle passages on the puncture site [26]. The difference of the predictors of TIVAD complication may be ascribed to the different techniques adopted in the two series: in our series the prevalent access was the US guided through internal jugular vein compared to the subclavicular access used in the Mansfield series.

The infection of the pocket was the most frequent late complications and the main cause of TIVAD removal in our series. Similarly, in the series described by Koch et al., this complication occurred in around 5% of all cases [5]. In fact, the infection of the pocket occurred more often after 20 weeks and after 60 weeks and this observation suggests that the postoperative infection is extremely rare and the causes are mainly related to its use (20 weeks peak) or lack of monthly maintenance (60 weeks peak). Curiously enough, in our series, hematologic malignancies were not more prone to infection and TIVAD extraction. The second most important complication, thrombosis, can occur in around 4.7 to 8.46% [27,28] but in our series it was much less frequent probably because of the regular rinsing of the catheter with heparin. Catheter malfunction can be caused mainly by the formation of a fibrin sheath that functions as a one-way valve in over 50% of all cases and by intraluminal thrombotic occlusion in 2-3%. Finally in our series, we did not observe any late complications such as: catheter rupture or migration, pinch-off syndrome, erosion with perforation of superior vein cava.

The main limit of this study is its retrospective design. Nevertheless, this limit was in part ridden over by the use of a prospectively collected database. The second limit of this study was the different sample size of the three groups. In fact, the first two groups are smaller than the last one. However, the overall sample size of the three groups was sufficient to make adequate comparison. Although our study provides a good definition of pro and cons of the different techniques a proper randomized controlled trial should be warranted to definitely determine the best TIVAD insertion technique.

## Conclusions

In conclusion, the results of our study demonstrate that ultrasound guided percutaneous technique is the safest and the most cost effective method for TIVAD insertion. In fact, the overall early complications rate was significantly lower for the ultrasound technique and, in particular, there was a significant reduction of the risk of pneumothorax. In order to reduce the immediate complication rate on TIVAD insertion, a real time US-guided technique should be preferred for vein identification and for puncture guidance and this technique may be assumed as standard care in case of TIVAD placement.

## Competing interests

The authors declare that they have no competing interests.

## Authors’ contributions

EG and MS gave substantial contributions to conception and design, to acquisition of data and to analysis and interpretation of data and they were involved in drafting the manuscript. AC gave substantial contributions to acquisition of data and interpretation of data and he was involved in critical revising for important intellectual content. BF gave substantial contributions to analysis and interpretation of data and he was involved in drafting the manuscript. MC, VM, RA, CC and SC gave substantial contributions to acquisition of data and they were involved in critical revising for important intellectual content. CC and MM gave substantial contributions to conception and design and interpretation of data and they were involved in drafting the manuscript. All authors read and approved the final manuscript.

## Pre-publication history

The pre-publication history for this paper can be accessed here:

http://www.biomedcentral.com/1471-2482/14/27/prepub
